# Spatial Distribution of Root and Crown Rot Fungi Associated With Winter Wheat in the North China Plain and Its Relationship With Climate Variables

**DOI:** 10.3389/fmicb.2018.01054

**Published:** 2018-05-25

**Authors:** Fei Xu, Gongqiang Yang, Junmei Wang, Yuli Song, Lulu Liu, Kai Zhao, Yahong Li, Zihang Han

**Affiliations:** Institute of Plant Protection, Henan Academy of Agricultural Sciences, Key Laboratory of Integrated Pest Management on Crops in Southern Region of North China, Ministry of Agriculture of the People's Republic of China, Zhengzhou, China

**Keywords:** root and crown rot of wheat, *Fusarium pseudograminearum*, the North China Plain, *Bipolaris sorokiniana*, *Rhizoctonia cerealis*

## Abstract

The distribution frequency of pathogenic fungi associated with root and crown rot of winter wheat (*Triticum aestivum*) from 104 fields in the North China Plain was determined during the period from 2013 to 2016. The four most important species identified were *Bipolaris sorokiniana* (24.0% from roots; 33.7% from stems), *Fusarium pseudograminearum* (14.9% from roots; 27.8% from stems), *Rhizoctonia cerealis* (1.7% from roots; 4.4% from stems), and *Gaeumannomyces graminis* var. *tritici* (9.8% from roots; 4.4% from stems). We observed that the recovered species varied with the agronomic zone. *Fusarium pseudograminearum* was predominant in regions 1 and 3, whereas *F. graminearum, F. acuminatum*, and *R. cerealis* were predominant in regions 2 and 4. The incidence of *F. pseudograminearum* and *R. cerealis* was significantly different between regions 1 and 4, while no significant association was found in the distribution of the other species and the agronomic zones. A negative correlation between the frequency of occurrence of *F. pseudograminearum* and mean annual precipitation during 2013–2016 (*r* = −0.71; *P* < 0.01) in the North China Plain and a positive correlation between the mean annual precipitation during 2013–2016 and the frequency of occurrence of *F. asiaticum* (*r* = 0.74; *P* < 0.01) were observed. Several *Fusarium* species were also found with low frequencies of ~2.1%−3.4 % (*F. graminearum, F. acuminatum*, and *F. sinensis*) and ~0.1%−1.3% (*F. equiseti, F. oxysporum, F. proliferatum, F. culmorum, F. avenaceum*, and *F. asiaticum*). In more than 93% of the fields, from the root and crown tissues of wheat, two or more root and crown rot species were isolated. The coexistence of *Fusarium* spp. and *B. sorokiniana* in one field (65.4%) or in individual plants (11.6%) was more common than for the other species combinations. Moreover, this is the first report on the association between *F. sinensis* and root and crown rot of wheat. Our results would be useful in the framing guidelines for the management of root and crown rot fungi in wheat in different agronomic zones of the North China Plain.

## Introduction

**Figure 1:**
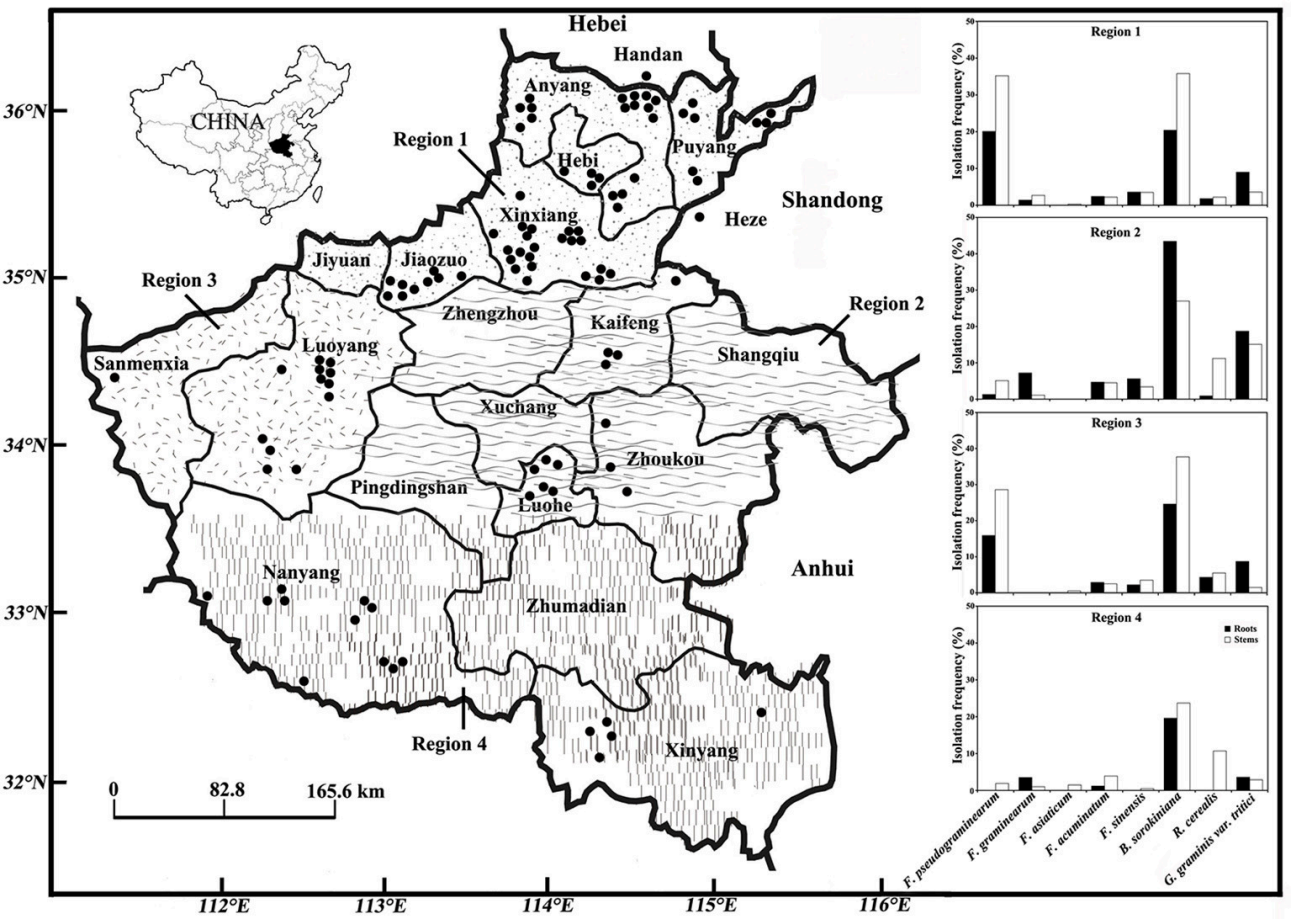
Isolation frequency of *Fusarium* spp., *Bipolaris sorokiniana, Rhizoctonia cerealis*, and *Gaeumannomyces graminis* var. *tritici* from symptomatic wheat roots and stems from the major wheat- producing areas in the North China Plain during 2013–2016. The area of scattered dots indicates the irrigated areas of northern Henan, southern Hebei and western Shandong (region 1) including Anyang, Hebi, Puyang, Xinxiang, Jiaozuo, Handan, and Heze; the area of wavy lines indicates the supplementary-irrigated areas of central Henan (region 2) including Kaifeng, Luohe, and Zhoukou; the area of dashed lines indicates the dry-farming areas of western Henan (region 3) including Sanmenxia and Luoyang; the area of vertical lines indicates the rain-fed areas of southern Henan (region 4) including Nanyang and Xinyang. The symbol “•”represents the sampling places of diseased wheat associated with root and crown rot fungi.

China has one of the largest wheat-planting areas (21 million ha), worldwide, and produces 87.7867 million tons of wheat, which is 19% of the total world production (National Statistical Year book 2014). The North China Plain is the major wheat producing area, accounting for 72% of the China's output. However, the production in this area is increasingly being effected by the root and crown rots of wheat, which cause whiteheads at the filling stage and reduce the crop yield (Li et al., 2012; Xu et al., 2014, 2016; Ji et al., 2016). The climatic conditions vary across the Yellow River, the Yangtze River, and the Haihe River of Henan in the North China Plain, the average annual temperature rang from 13 to 16°C, the rainfall from 300 to 1,100 mm, and the elevation from 23.2 to 2413.8 m. The rainfall mostly occurs in summer (June, July, and August) and is the lowest in July. The wheat-growing areas of Henan and its neighboring provinces in the North China Plain can be divided into four regions according to the soil type and climate (Hu and Yin, 2014); these include the irrigated areas of northern Henan, southern Hebei, and western Shandong (region 1), the supplementary-irrigated areas of central Henan (region 2), the dry-farming areas of western Henan (region 3), and the rain-fed areas of southern Henan (region 4) (Figure 1, Table 1). As the incidence of root and crown rot diseases of wheat are reported to be different in the four agronomic zones and several pathogenic fungi can cause the same disease in wheat, the distribution of predominant species associated with root and crown rot and their relationship with the climate variables have been important issues for research in the North China Plain.

**Table 1:** Wheat production and physio-climatic variation in the 2013–2016 survey sites in Henan.

**City^a^**	**Region**	**Area under wheat cultivation (1,000 ha)**	**Elevation (m)**	**2013**		**2014**		**2015**		**2016**	
				**MAT^b^ (°C)**	**AP^c^ (mm)**	**MAT (°C)**	**AP (mm)**	**MAT (°C)**	**AP (mm)**	**MAT (°C)**	**AP (mm)**
Anyang	1	309.4	48.8–462.8	14.3	458.2	14.8	516.4	14.4	563.6	14.9	647.9
Puyang		220.2	42.4–59.9	14.0	476.8	14.5	474.2	14.1	543.1	14.7	539.5
Hebi		88.0	56.4–73.1	14.1	348.5	14.8	407.7	14.3	459.1	14.8	633.6
Xinxiang		341.8	71.1–87.1	14.8	422.5	14.8	499.1	14.3	511.7	15.0	703.5
Jiaozuo		142.9	100.9–109.3	15.5	419.0	15.6	509.4	15.3	550.3	15.8	594.8
Kaifeng	2	301.1	63.4–73.2	15.7	335.3	15.9	508.1	15.4	583.6	16.0	655.6
Luohe		142.6	64.6–67.2	15.2	716.8	15.2	628.1	14.9	738.6	15.5	712.2
Zhoukou		678.4	44.4–50.9	16.6	739.2	16.5	778.9	16.3	692.5	16.7	799.1
Sanmenxia	3	71.1	410.1–465.0	15.1	338.5	14.6	605.2	14.3	722.8	14.9	590.2
Luoyang		251.2	115.2–318.3	15.5	518.6	14.5	674.1	14.2	594.8	14.8	562.7
Nanyang	4	677.7	100.8–159.5	16.5	649.9	15.7	767.1	15.5	691.3	16.2	820.8
Xinyang		314.6	52.4–90.2	16.6	952.0	16.2	1111.9	16.1	957.4	16.6	1317.8
**AGRONOMIC ZONE**											
Region 1		1102.3	42.4–462.8	14.5	425.0	14.9	481.4	14.5	525.6	15.0	623.9
Region 2		1122.1	44.4–73.2	15.8	597.1	15.9	638.4	15.5	671.6	16.1	722.3
Region 3		322.3	115.2–465.0	15.3	428.6	14.6	639.7	14.3	658.8	14.9	576.5
Region 4		992.3	52.4–159.5	16.6	801.0	16.0	939.5	15.8	824.4	16.4	1069.3

a *Anyang, Puyang, Hebi, Xinxiang, and Jiaozuo are in the irrigated areas of northern Henan, southern Hebei, and western Shandong (region 1); Kaifeng, Luohe, and Zhoukou are in the supplementary-irrigated areas of central Henan (region 2); Sanmenxia and Luoyang are in the dry-farming areas of western Henan (region 3); Nanyang and Xinyang are in the rain-fed areas of southern Henan (region 4)*.

b *MAT = mean annual temperature*,

c *AP = annual precipitation*.

The root and crown rots are important diseases of cereals, worldwide, and are found to occur in the major winter wheat growing regions in China (Zhang et al., 2015). The infection of the root and crown causes the vascular system to constrict, which limits the uptake and transport of water, causing whiteheads at the filling stage (Cook, 2010; Poole et al., 2013). There are four important diseases of the root in China as well as in other countries of the world, namely Fusarium root and crown rot (FCR), common root and foot rot (CRR), sharp eyespot, and take-all (Burgess et al., 2001; Fernandez et al., 2009; Cook, 2010). The recognition of the role of soil-borne pathogens as factors limiting the production of wheat in most of the areas of the North China Plain is problematic. The identification and quantification of root and crown rot diseases involves more laborious procedures compared to those involved in simple visual observations.

Fusarium root and crown rot is caused by a complex of species that includes *Fusarium pseudograminearum* (O'Donnell and T. Aoki), *F. graminearum* (Schwabe), and *F. culmorum* (Wm. G. Smith) Sacc. (Leslie and Summerell, 2006; Cook, 2010; Moya-Elizondo, 2013). Four other *Fusarium* species, including *F. acuminatum* (EIIis and Everh.), *F. avenaceum* (Fr.:Fr.) Sacc., *F. crookwellense* (L. W. Burgess, P. E. Nelson, and Toussoun), and *F. poae* (Peck) Wollenw., also infect the wheat roots, lower culms, and leaf sheaths but are less virulent and more environmentally or geographically restricted than the three species mentioned above (Smiley and Patterson, 1996; Paulitz et al., 2002; Cook, 2010; Moya-Elizondo et al., 2011). Several other genera also cause wheat root and crown rot, including the causal agents of CRR, *Bipolaris sorokiniana* (Sacc.) Shoemaker (= *Cochliobolus sativus*); the causal agents of take-all, *Gaeumannomyces graminis* (Sacc.) Arx and D. L. Oliver, and *G. graminis* var. *tritici* J. Walker and the causal agents of sharp eyespot, *Rhizoctonia* spp. The *Rhizoctonia* spp. associated with wheat root and crown rot disease include *Rhizoctonia solani* (J. G. Kühn), *R. oryzae* (Ryker and Gooch), and *R. cerealis* (E. P. Hoeven).

Several surveys of FCR-causing organisms have been conducted in the dryland wheat-growing regions around the world (Cook, 2010). Although the causal agents for FCR are frequently a complex of *F. pseudograminearum* and *F. culmorum*, other pathogens capable of causing crown infection include *F. graminearum, F. acuminatum, F. avenaceum*, and *Microdochium nivale* (Fr.:Fr.) Samuels and I. C. Hallett [syn. *F. nivale* Ces. ex Berl. and Voglino; teleomorph *Monographella nivalis* (Schaffnit) E. Müll.]. It is widely accepted that *F. pseudograminearum* is the dominant species responsible for crown rot in the arid zones of the Pacific Northwest (PNW) and Australia (Smiley and Patterson, 1996; Backhouse and Burgess, 2002; Backhouse et al., 2004; Poole et al., 2013). *Fusarium culmorum* was reported to be the predominant species in eastern Victoria and South Australia (Smiley and Patterson, 1996; Backhouse and Burgess, 2002); and *F. graminearum* was reported to be virulent in eastern Australia and southern Europe. However, other *Fusarium* spp. were more restricted, geographically. *Fusarium acuminatum, F. avenaceum*, and *M. nivale* were reported to be prevalent in southern Australia (Williams et al., 2002), the PNW (Backhouse and Burgess, 2002; Paulitz et al., 2002), and Europe, respectively.

Besides *Fusarium* spp., *B. sorokiniana* associated with CRR was reported to be prevalent in the arid and semiarid regions of western USA and in Jiangsu province of China, and was found to be a primary component of the cereal foot rot complex in Oregon and Washington in the USA (Smiley and Patterson, 1996; Li et al., 2011). *Gaeumannomyces graminis* var. *tritici* is widely distributed in Australia, Europe, South Africa, Japan, Brazil, Chile, Argentina, China, and most of the North America. The Rhizoctonia root rot and bare patch are caused by *R. solani*, which causes lesions and pruning of the seminal and crown roots, particularly in the rained cereal production systems in Australia and the PNW (Paulitz et al., 2002). *Rhizoctonia oryzae* also causes pre-emergence and post-emergence damping-off, but this is not common with *R. solani* in the PNW (Paulitz et al., 2002). *Rhizoctonia cerealis* has been reported to be widespread in China and Turkey where is an economically important crown pathogen of wheat (Tunali et al., 2008; Chen et al., 2009).

The distribution of species and root and crown rot disease in certain wheat-growing regions is influenced by the climate (Backhouse and Burgess, 2002; Leslie and Summerell, 2006; Bentley et al., 2009; Cook, 2010; Moya-Elizondo, 2013). Among the FCR pathogens, the distribution of *F. pseudograminearum* isolates was observed to be inversely proportional to the rainfall within a certain range (Smiley and Patterson, 1996; Poole et al., 2013). *Fusarium pseudograminearum* FCR was more widespread during the low-rainfall years in the PNW of the United States and in the low rainfall (250–500 mm) areas of Australia. On the other hand, *F. culmorum* was predominant in the high rainfall areas of eastern Australia (>500 mm) and in the cooler and higher altitude areas of Idaho (Backhouse et al., 2004; Strausbaugh et al., 2004; Smiley et al., 2005). The incidence of FCR was reported to differ for different years and wheat varieties, and *Fusarium* population dynamics were affected by the climate (Smiley and Patterson, 1996; Smiley et al., 2005; Smiley and Yan, 2009). The common root rot is most severe when plants are drought-stressed (Stein, 2010). However, take-all is most severe in wet areas or during the wet years and in irrigated fields (Paulitz, 2010).

Surveys of *Fusarium* spp. causing wheat crown rot have been conducted throughout the major winter wheat growing regions of China and the frequency of FCR in Henan and Hebei has been recorded (Li et al., 2012; Zhang et al., 2015; Ji et al., 2016). Li et al. (2012) first reported *F. pseudograminearum*, causing FCR, from the Henan Province in China and it was shown to be the predominant FCR pathogen in Hebei (Ji et al., 2016). However, *F. asiaticum* and *F. graminearum*, causing wheat crown rot were predominant in Henan, Hebei and Shangdong in the North China Plain during the period from 2009 to 2013 (Zhang et al., 2015). The common root rot caused by *B. sorokiniana* has been reported in Heilongjiang and Jiangsu provinces of China (Zhang et al., 1988; Li et al., 2011), but not in the North China Plain. *Gaeumannomyces graminis* var. *tritici* and *R. cerealis* have also been reported to be widespread in China (Chen et al., 2009; Xu et al., 2014).

Although previous surveys of pathogenic fungi associated with root and crown rot disease have been conducted in Henan, Hebei, and Shandong provinces of the North China Plain (Li et al., 2011, 2012; Xu et al., 2014; Zhang et al., 2015), the distribution of *F. pseudograminearum* and *B. sorokiniana* in the North China Plain is not known with certainity as to whether there is a coexistence of different species in one field or in an individual plant and whether there are significant differences in the predominance of species associated with root and crown rot between different agronomic zones. Therefore, the distribution of root and crown rot fungi causing wheat white heads was assessed during the period from 2013 to 2016 across the four regions of Henan province in the North China Plain. The objectives of the present study are (i) to determine the distribution of pathogenic fungi associated with root and crown rot in different agronomic zones of Henan and its neighboring provinces, in one field or in individual plant, (ii) to ascertain if the predominant species recovered from an area correlated with the climate variables.

## Materials and methods

### Survey of ecological and regional zones and sampling

This study was performed in four representative wheat-growing regions of Henan and its neighboring cities of Handan in Hebei and Heze in Shandong (Figure 1, Table 1). The wheat fields were selected randomly with respect to the selected region (2–3 fields per village, 2–5 villages per county, 2–5 counties in each region) and severity of disease. The selected fields were at least 5 km apart. The area of each field was in the 2,000–6,667 m^2^ range. The samples were collected from five sites in the field in a zig-zag pattern (Fang, 1998). Each sampling site was 1–2 m^2^, and 10–30 m apart. Ten plants were collected from each site, and the ratio of whiteheads in each sample of 10 plants was recorded. Thus, a total of 50 plants were collected from each field. The fields were geo-referenced using a Magellan global positioning system (GPS) meter and numbered accordingly. If a selected field exhibited a low infection rate, the diseased plants were collected from wherever they were found. The diseased stems were collected from 104 fields in 15 cities from May 5 to May 30 during the period from 2013 to 2016 (Feekes growth stages 11.1 Large, 1954).

### Isolation, culture, and identification of species

The diseased stems collected from each field were washed thoroughly under tap water. Three diseased crown or stem sections (1–1.5 cm) and three diseased root sections (1–1.5 cm) were collected from each plant (six sections per plant). These sections were surface-sterilized sequentially with 70% ethanol for 10 s and 3% sodium hypochlorite for 1–2 min, rinsed thrice with distilled water, and then dried on a sterile paper towel. The samples were plated onto potato dextrose agar (PDA) (200 g of peeled potato, 20 g of dextrose, and 20 g of agar in 1,000 mL distilled water) medium containing 150 μg/mL streptomycin and 75 μg/mL penicillin (six sections from one stem per PDA plate). If the roots did not show any infection, only three crown or stem sections were selected from the plant. The plates were incubated at 25°C for 3–7 days under a day/night photoperiod of 12/12 h. The degree of infection was recorded and the isolates were then transferred to fresh PDA plates.

The isolates of *Fusarium* spp. were purified using the single spore isolation protocol described by Xu et al. (2016) and were initially identified morphologically on synthetic nutrient agar (SNA) and carnation leaf agar (CLA) (Leslie and Summerell, 2006). Other fungi were identified using keys based on their colony, hyphal, or conidial morphologies.

The PCR amplification and sequencing of *EF-1*α from 1120 isolates of *Fusarium* spp. were conducted using the primer pair, EF1 and EF2 (O'Donnell et al., 2000, 2004; Proctor et al., 2009). The internal transcribed spacer (ITS) sequences of other representative species were amplified and sequenced with the primer pair, ITS1 and ITS4 (White et al., 1990). The PCR products were sequenced by Shanghai Sangon Biological Engineering Co., Ltd. The DNA sequences were aligned and adjusted using DNAStar-SeqMan software (http://www.dnastar.com/). The phylogenetic relationships were inferred with MEGA7.0.18 (http://www.megasoftware.net/megamacBeta.php) using the maximum-likelihood method, with 1,000 bootstrap replicates. The best-fit model of molecular evolution was selected based on the estimation of Bayesian Information Criterion scores.

### Environmental characterization

The climate data were downloaded from the database developed by the Henan Meteorological Bureau (http://www.henanqx.gov.cn/) and were selected from the data available from the weather forecast station nearest to the sample field in each county. These data include six variables for 4-year norms for the period from 2013 to 2016. A correlation analysis of these six variables indicated that most of them were highly correlated. As a result, a subset including mean annual temperature (MAT), mean annual precipitation (MAP), mean temperature in the coldest month (MTCM), and mean temperature in the warmest month (MTWM) were the only variables in the analyses described below.

### Virulence assays

To ensure the representation of a range of geographic origin, the pathogenicity of 35 isolates of *Fusarium* spp. (five isolates each of *F. pseudograminearum, F. graminearum, F. acuminatum, F. sinensis* (Z. Zhao and G. Lu), *F. equiseti* (Corda) Sacc., *F. oxysporum* (Schlechtendahl emend. Snyder and Hansen), and *F. proliferatum* (Matsushima) Nirenberg) and five isolates of *Bipolaris sorokiniana* was tested on wheat seedlings (*Triticum aestivum* cultivar “Zhengmai 366”) in a glasshouse under 12/12 h (day/night) photoperiod at a temperature of 25/15°C, and relative humidity of 60/80% (±5%) for 35 days using the method described by Mitter et al. (2006).

To prepare the inoculum of macroconidia, the isolates were grown on PDA plates for 3–6 days at 25°C in the dark, and then 10 pieces of PDA colonized by the fungus (0.25 cm^2^) were placed in 100 mL mung bean liquid medium (4%) and cultured in 250 mL Erlenmeyer flasks at 25°C on an orbital shaker at 150 rpm for 5 days, and then filtered using conventional microscope lens paper (Zhejiang Province Wenzhou Handicraft Factory). The mung bean liquid medium (4%) was prepared by boiling 40 g of green beans in distilled water until the pericarp started to crack-open; the extract was filtered through several layers of cheesecloth and the volume was made up to 1 L with distilled water. The medium was autoclaved for 20 min at 121°C. The concentration of conidia was determined using a hemocytometer and the suspension was then diluted to 1 × 10^6^ spores/mL for seedlings inoculation (Mitter et al., 2006).

The method described by Mitter et al. (2006) was amended for inoculation of seedling stem. Ten wheat seedlings were grown in sterilized soil (50% natural soil and 50% sand, v/v) in plastic pots (diameter = 10 cm) in a glasshouse under 12/12 h (day/night) photoperiod at 25/15°C. Ten days after the emergence, each seedling of “Zhengmai 366” (*Triticum aestivum*) was inoculated with a 10-μL droplet of a suspension of 1 × 10^6^ spores/mL. One isolate was used to inoculate 30 seedlings (10 seedlings per block). The inoculated seedlings were incubated at near-saturated relative humidity in darkness for 48 h, and then transferred to a glasshouse under 12/12 h (day/night) photoperiod at 25/15°C and a relative humidity of 60/80% (±5%) for 35 days. The degree of disease was rated with one of the five grades, modified from Zhang et al. (2015): 0 = no disease; 1 = trace to 10% of the first leaf sheath discolored; 2 = 11%-25% of first leaf sheath discolored; 3 = 26%-50% of the first leaf sheath discolored; 4 ≥ 50% of the first leaf sheath discolored or obviously necrotic second leaf sheath; 5 = third leaf sheath obviously necrotic or entire plant severely to completely necrotic.

### Statistical analysis

A one-way factorial analysis of variance (ANOVA) (SAS Institute, Cary, NC, USA, Version 8.0, 1999) was used to determine the differences in the proportions of species associated with specific regions or agro-zones and virulence of isolates of root and crown rot fungi. The means for different *Fusarium* spp. and *B. sorokiniana* were separated using Fisher's Protected Least Significant Difference Test (*P* < 0.05). Pearson correlations were calculated to estimate the relatedness among the four-year averaged continuous climate and geographic variables. The correlations were calculated between MAT, MAP, MTCM, MTWM and the predominant species recovered in an area. The isolation frequency of the predominant species recovered in an area or a field was evaluated as follows: 100 × (the total number of isolates belonging to a specific species from roots and stems in an area or a field/ the total number of roots and stems in an area or a field).

## Results

### Isolation and geographical distribution of species

**Figure 2:**
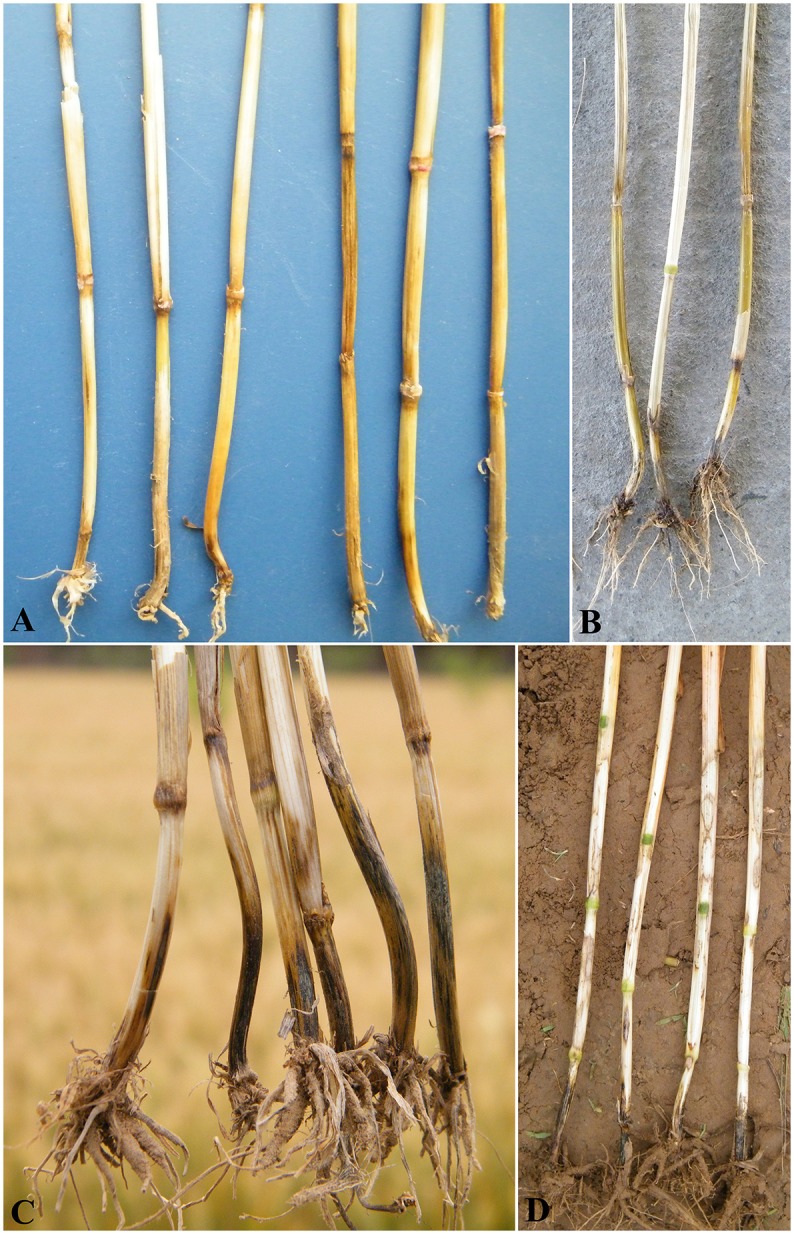
Brown discoloration of lower stem internodes and dark discoloration of nodes caused by *Fusarium pseudograminearum* and *Bipolaris sorokiniana* in individual plants **(A)**, Brown discoloration of lower stem internodes caused by *F. pseudograminearum* and blackening of the basal stem caused by *Gaeumannomyces graminis* var. *tritici* also exist alone and coexist in individual plants in the same field **(B)**, Brown discoloration of lower stem internodes caused by *B. sorokiniana* and blackening of the basal stem caused by *G. graminis* var. *tritici* in individual plants **(C)** and sharp eyespot on the leaf sheath caused by *Rhizoctonia cerealis* and blackening of the basal stem caused by *G. graminis* var. *tritici* in individual plants **(D)**.

**Figure 3:**
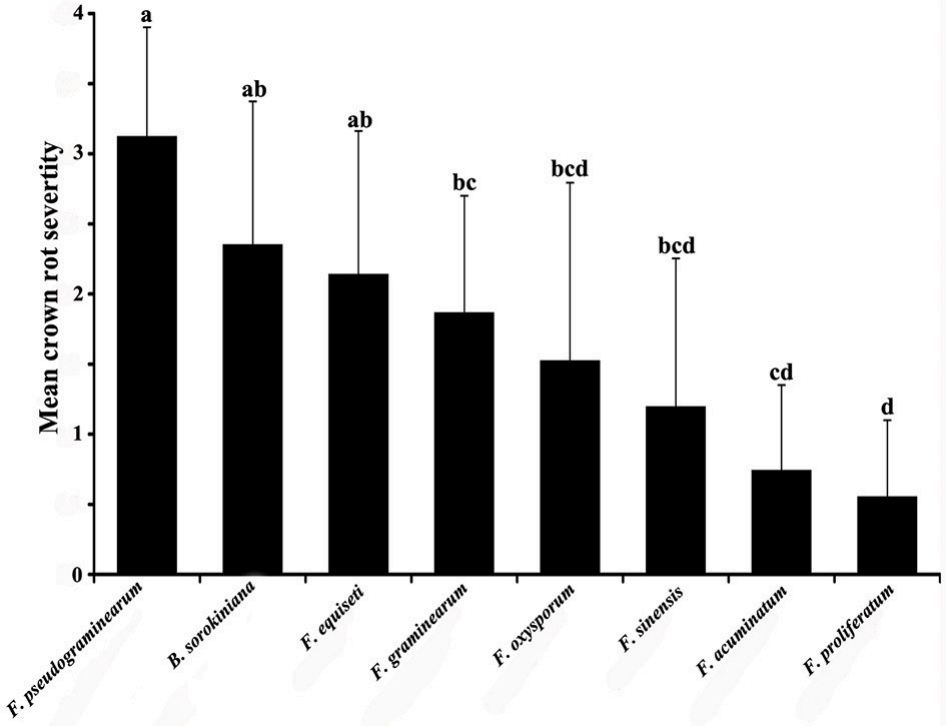
Variation in pathogenicity of 35 isolates of *Fusarium* spp. (five isolates each of *Fusarium pseudograminearum, F. graminearum, F. acuminatum, F. sinensis, F. equiseti, F. oxysporum*, and *F. proliferatum*) and five isolates of *Bipolaris sorokiniana* on wheat seedlings (*Triticum aestivum* cultivar “Zhengmai 366”) in a glasshouse with a day/night photoperiod of 12/12 h at a temperature of 25/15°C and relative humidity of 60/80 (±5) % at 35 days after inoculation. Means followed by the same letter are not significantly different (*P* = 0.05).

**Figure 4:**
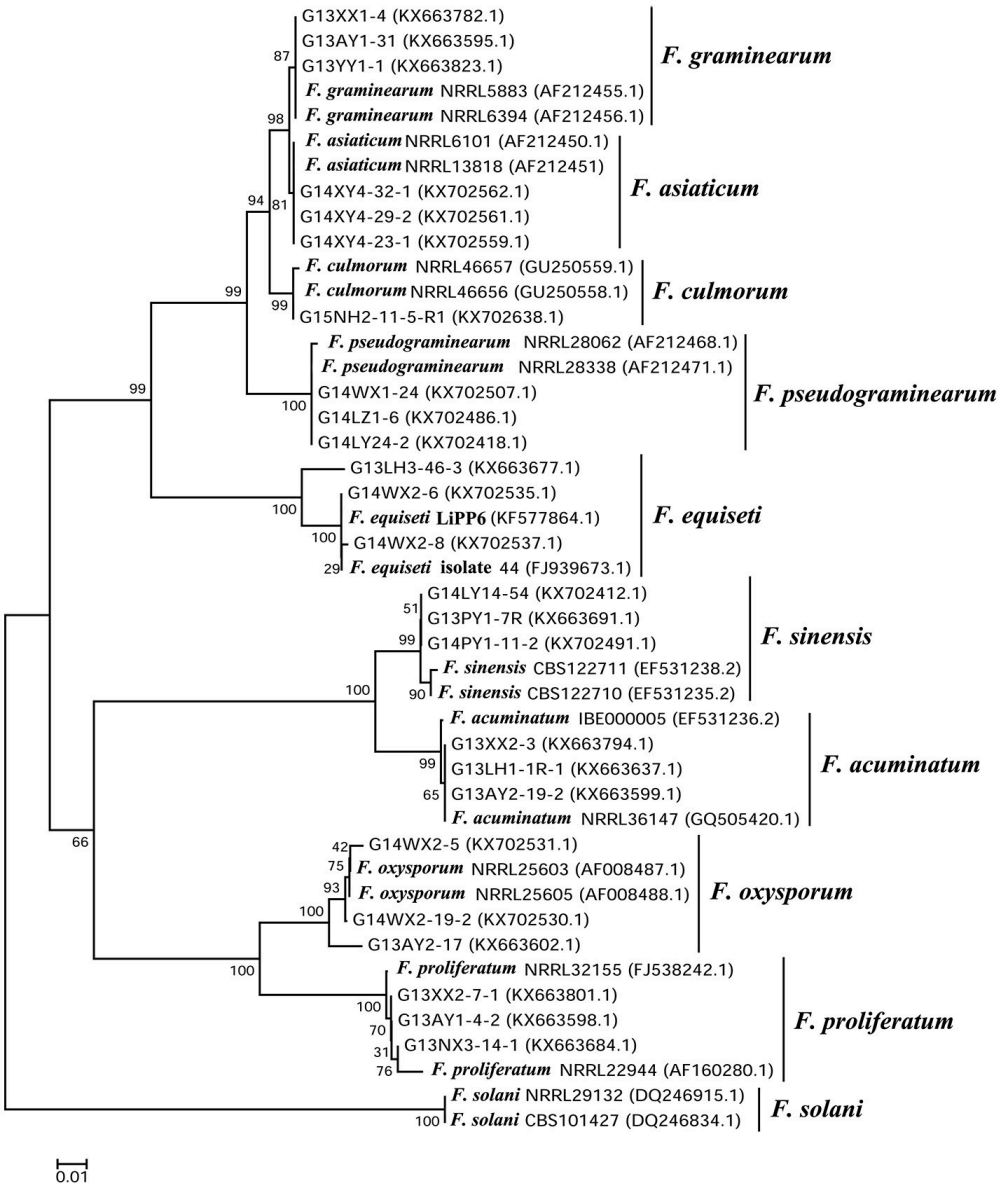
Phylogeny of *Fusarium* spp. based on the translation elongation factor 1a (*EF-1*α) gene region (Maximum Likelihood). The bootstrap values (percentage, based on 1,000 replications) are shown on the branches. Some representative strains of *Fusarium* spp. from this study were selected for the phylogenetic tree. The remaining isolates were retrieved from the National Center for Biotechnology Information database. *F. solani* was used as an outgroup.

Based on morphological and molecular identification, 1120 strains of *Fusarium* spp., 1004 of *B. sorokiniana*, 107 of *R. cerealis*, and 238 of *G. graminis* var. *tritici* were collected from 104 fields in the North China Plain during the 2013–2016 growing seasons (Tables 2–4; Figures 1, 2). Among the different strains of *Fusarium* spp., the sequences of 871 representative strains were deposited in the National Center for Biotechnology Information and accession numbers were obtained (Table S3). Based on the phylogenetic analysis of *EF-1*α sequences, 10 *Fusarium* species were identified in our study on FCR in wheat roots and stems (Figures 3, 4, and Figure S1).

**Table 2:** Incidence of *Fusarium* spp., *Bipolaris sorokiniana, Rhizoctonia cerealis*, and *Gaeumannomyces graminis* var. *tritici* isolated from wheat roots from each geographical region during the period from 2013 to 2016.

**City^b^**	**Region**	**No. of** **roots**	**Percentage of roots containing the species**^a^ **(%)**											
			***pg***	***g***	***ac***	***s***	***e***	***o***	***pr***	***c***	***av***	***Bs***	***Rc***	***Ggt***
Anyang	1	274	23.0	1.8	2.6	2.6	0.4	1.5	0.0	0.4	0.0	19.3	0.0	5.5
Puyang		141	15.6	0.7	2.1	10.6	0.7	0.7	0.7	0.0	0.0	19.9	5.7	5.7
Hebi		95	25.3	1.1	2.1	5.3	0.0	0.0	1.1	0.0	0.0	55.8	0.0	9.4
Xinxiang		344	14.2	1.2	3.8	1.7	0.9	0.6	1.2	0.0	0.0	16.6	3.2	18.0
Jiaozuo		196	28.6	2.0	0.5	3.1	2.0	1.0	1.5	0.0	0.0	11.7	0.0	0.5
Handan		10	10.0	0.0	0.0	0.0	0.0	0.0	0.0	0.0	0.0	30.0	0.0	0.0
Heze		10	0.0	0.0	0.0	0.0	0.0	0.0	0.0	0.0	0.0	10.0	0.0	10.0
Kaifeng	2	8	37.5	0.0	12.5	0.0	0.0	0.0	0.0	0.0	0.0	25.0	0.0	0.0
Luohe		183	0.0	9.3	5.5	6.6	0.0	0.0	0.5	0.0	0.0	48.6	0.6	24.0
Zhoukou		44	0.0	0.0	0.0	2.3	0.0	0.0	0.0	0.0	0.0	25.0	2.3	0.0
Sanmenxia	3	13	0.0	0.0	30.8	7.7	0.0	0.0	0.0	0.0	7.7	0.0	0.0	69.2
Luoyang		125	17.6	0.0	0.0	1.6	0.8	2.4	0.0	0.0	0.0	27.2	4.8	2.4
Nanyang	4	133	0.0	4.5	0.0	0.0	0.0	1.5	1.5	0.0	0.0	22.6	0.0	0.0
Xinyang		36	0.0	0.0	5.6	0.0	0.0	0.0	0.0	0.0	0.0	8.3	0.0	16.7
**AGRONOMIC ZONE**														
Region 1		1,070	20.1	1.4	2.4	3.6	0.9	0.9	0.9	0.1	0.0	20.4	1.8	9.0
Region 2		235	1.3	7.2	4.7	5.6	0.0	0.0	0.4	0.0	0.0	43.4	0.9	18.7
Region 3		138	15.9	0.0	2.9	2.2	0.7	2.2	0.0	0.0	0.7	24.6	4.3	8.7
Region 4		169	0.0	3.5	1.2	0.0	0.0	1.2	1.2	0.0	0.0	19.6	0.0	3.6
Total		1,612	14.9	2.4	2.7	3.4	0.6	0.9	0.7	0.1	0.1	24.0	1.7	9.8

a *Fusarium pseudograminearum (pg), F. graminearum (g), F. acuminatum (ac), F. sinensis (s), F. equiseti (e), F. oxysporum (o), F. proliferatum (pr), F. culmorum (c), F. avenaceum (av), Bipolaris sorokiniana (Bs), Rhizoctonia cerealis (Rc), and Gaeumannomyces graminis var. tritici (Ggt)*.

b *Handan city is in Hebei province, Heze city is in Shandong province, and the other cities are in Henan province. Anyang, Hebi, Puyang, Xinxiang, Jiaozuo, Handan and Heze are in the irrigated areas of northern Henan, southern Hebei and western Shandong (region 1); Kaifeng, Luohe and Zhoukou are in the supplementary-irrigated areas of central Henan (region 2); Sanmenxia and Luoyang are in the dry-farming areas of western Henan (region 3); Nanyang and Xinyang are in the rain-fed areas of southern Henan (region 4)*.

*Fusarium pseudograminearum, B. sorokiniana*, and *G. graminis* var. *tritici* were the predominant pathogens recovered from the wheat root samples, with isolation frequencies of 14.9, 24, and 9.8% (N_roots_ = 1,612 plants), respectively, whereas *F. graminearum, F. acuminatum, F. sinensis, F. equiseti, F. oxysporum, F. proliferatum, F. culmorum, F. avenaceum*, and *R. cerealis* were the minor pathogens identified, with isolation frequencies of 2.4, 2.7, 3.4, 0.6, 0.9, 0.7, 0.1, 0.1, and 1.7%, respectively (Table 2). Similarly, *F. pseudograminearum* and *B. sorokiniana* were also the major species found in the stems in these areas, with isolation frequencies of 27.8 and 33.7% (N_stems_ = 1831 plants), respectively, and *F. graminearum, F. asiaticum, F. acuminatum, F. sinensis, F. equiseti, F. oxysporum, F. proliferatum, R. cerealis*, and *G. graminis* var. *tritici* were the minor pathogens with isolation frequencies of 2.1, 0.4, 2.6, 3.2, 1.3, 0.5, 0.7, 4.4, and 4.4%, respectively (Table 3).

**Table 3:** Incidence of *Fusarium* spp., *Bipolaris sorokiniana, Rhizoctonia cerealis*, and *Gaeumannomyces graminis* var. *tritici* isolated from wheat stems from each geographical region during the period from 2013 to 2016.

**City**	**Region**	**No. of** **stems**	**Percentage of stems containing the species**^a^ **(%)**										
			***pg***	***g***	***as***	***ac***	***s***	***e***	***o***	***pr***	***Bs***	***Rc***	***Ggt***
Anyang	1	302	36.4	3.3	0.0	1.3	2.0	1.3	0.7	1.3	26.8	4.3	1.7
Puyang		158	29.7	2.5	2.5	0.6	3.2	0.0	0.0	0.6	25.9	7.0	1.3
Hebi		103	40.8	0.0	0.0	1.0	3.9	0.0	0.0	0.0	78.6	0.0	2.9
Xinxiang		433	29.1	2.8	0.0	4.4	3.7	0.9	0.9	1.2	38.6	0.2	7.9
Jiaozuo		227	46.3	3.5	0.0	0.9	5.7	4.8	0.0	0.9	27.3	1.3	0.4
Handan		14	42.9	0.0	0.0	0.0	0.0	0.0	0.0	0.0	64.3	0.0	0.0
Heze		10	30.0	0.0	0.0	0.0	0.0	0.0	0.0	0.0	50.0	0.0	0.0
Kaifeng	2	29	27.6	0.0	0.0	3.4	0.0	0.0	0.0	0.0	17.2	37.9	0.0
Luohe		102	1.0	2.0	0.0	5.9	5.9	0.0	0.0	0.0	32.4	4.9	17.6
Zhoukou		47	0.0	0.0	0.0	2.1	0.0	0.0	0.0	0.0	21.3	8.5	19.1
Sanmenxia	3	14	0.0	0.0	0.0	35.7	28.6	0.0	0.0	0.0	21.4	0.0	14.3
Luoyang		185	30.8	0.0	0.5	0.0	1.6	1.6	1.1	0.0	38.9	5.9	0.5
Nanyang	4	160	2.5	1.3	0.0	1.3	0.6	0.0	0.0	0.6	26.3	11.3	0.0
Xinyang		47	0.0	0.0	6.4	12.8	0.0	2.1	2.1	0.0	14.9	8.5	12.8
**AGRONOMIC ZONE**													
Region 1		1,247	35.2	2.7	0.3	2.2	3.5	1.5	0.5	1.0	35.8	2.2	3.6
Region 2		178	5.1	1.1	0.0	4.5	3.4	0.0	0.0	0.0	27.0	11.2	15.1
Region 3		199	28.6	0.0	0.5	2.5	3.5	1.5	1.0	0.0	37.7	5.5	1.5
Region 4		207	1.9	1.0	1.5	3.9	0.5	0.5	0.5	0.5	23.7	10.7	2.9
Total		1,831	27.8	2.1	0.4	2.6	3.2	1.3	0.5	0.7	33.7	4.4	4.4

a *Fusarium asiaticum (as), the other species see Table 2. for abbreviations*.

*Fusarium* spp., *B. sorokiniana, R. cerealis*, and *G. graminis* var. *tritici* could be found in each of the four agronomic zones in the survey, but the incidence of individual species varied among these zones. The incidence of root and crown rot fungi was calculated as the percentage of isolation frequency from roots or stems in each region. The incidence of *F. pseudograminearum* was significantly different between region 1 (35.2%, N_stems_ = 1247 plants) and 4 (1.9%, N_stems_ = 207 plants) (*t* = 4.84, *df* = 56, *P* < 0.0001; Figure 1), whereas no significant associations were found in the proportion of the other *Fusarium* spp. with the different agronomic zones. In contrast, the incidence of *R. cerealis* was significantly different between region 1 (2.2%, N_stems_ = 1247 plants) and region 4 (10.7%, N_stems_ = 207 plants) (*t* = −1.85, *df* = 16, *P* < 0.05). However, there were no significant associations in the proportion of *B. sorokiniana* and *G. graminis* var. *tritici* with the different agronomic zones (Figure 1).

### Species diversity in single fields and in individual plants

In more than 93% of the fields were retrieved two or more of the root and crown rot species isolated from the root and crown tissues of wheat. With respect to individual species, 49% of the fields had *F. pseudograminearum*, 20.2% had *F. graminearum*, 3.8% had *F. asiaticum*, 26% had *F. acuminatum*, 29.8% had *F. sinensis*, 82.7% had *B. sorokiniana*, 21.2% had *R. cerealis*, and 27.9% had *Gaeumannomyces graminis* var. *tritici* (Table 4 and Table S1). The different *Fusarium* spp. were observed to occurr alone but often coexisted in a single field (45.2%, N_fields_ = 104) and even within individual plants (1.7%, N_plants_ = 1902) (Tables S1, S2). For example, both *F. pseudograminearum* and *F. graminearum* were present in 11.5% of the fields (N_fields_ = 104), whereas *F. pseudograminearum*, together with *F. acuminatum, F. sinensis*, and *F. asiaticum*, were present in the same field, with frequencies of 9.6, 10.6, and 2.9%, respectively (Table S1). Similarly, *F. graminearum* and *F. acuminatum* were present in 5.8% of the fields (N_fields_ = 104), whereas *F. graminearum*, together with *F. sinensis* and *F. asiaticum*, were present in the same field, with frequencies of 7.7 and 1.0%, respectively (Table S1).

**Table 4:** Field incidence of fungal species recovered from symptomatic wheat plants collected from wheat fields in 14 cities of China from 2013 to 2016.

**City**	**Region**	**No. of fields**	**Percentage of fields with the species**^a^ **present (%)**											
			***Fspp***	***Bs***	***Rc***	***Ggt***	***Fspp+Bs***	***Fspp+Rc***	***Fspp+Ggt***	***Bs+Rc***	***Bs+Ggt***	***Rc+Ggt***	***Fspp+Bs+Rc***	***Fspp+Bs+Ggt***
Anyang	1	17	88.2	88.2	5.9	41.2	76.5	0.0	23.5	5.9	35.3	0.0	0.0	23.5
Puyang		8	87.5	62.5	25.0	37.5	50.0	12.5	25.0	25.0	37.5	37.5	12.5	0.0
Hebi		4	100.0	75.0	0.0	50.0	75.0	0.0	50.0	0.0	50.0	0.0	0.0	50.0
Xinxiang		22	86.4	90.9	13.6	36.4	77.3	13.6	27.3	13.6	31.8	4.5	9.1	18.2
Jiaozuo		9	100.0	100.0	11.1	11.1	100.0	11.1	11.1	11.1	11.1	11.1	11.1	0.0
Handan		1	100.0	100.0	0.0	0.0	100.0	0.0	0.0	0.0	0.0	0.0	0.0	0.0
Heze		1	100.0	100.0	0.0	100.0	100.0	0.0	100.0	0.0	100.0	0.0	0.0	100.0
Kaifeng	2	4	75.0	75.0	25.0	0.0	50.0	0.0	0.0	25.0	0.0	0.0	0.0	0.0
Luohe		6	66.7	83.3	33.3	33.3	50.0	33.3	0.0	16.7	33.3	0.0	16.7	0.0
Zhoukou		3	66.7	100.0	66.7	33.3	66.7	33.3	33.3	66.7	33.3	33.3	33.3	33.3
Sanmenxia	3	1	100.0	100.0	0.0	100.0	100.0	0.0	100.0	0.0	100.0	0.0	0.0	100.0
Luoyang		12	50.0	83.3	25.0	25.0	41.7	0.0	0.0	25.0	25.0	8.3	8.3	0.0
Nanyang	4	11	54.5	63.6	36.4	0.0	36.4	18.2	0.0	27.3	0.0	0.0	18.2	0.0
Xinyang		5	60.0	60.0	60.0	20.0	60.0	40.0	20.0	40.0	20.0	0.0	40.0	20.0
Total		104	77.9	82.7	21.2	27.9	65.4	11.5	18.3	18.3	26.0	6.7	10.6	13.5

a *Fusarium spp. (Fspp); Fusarium spp. including Fusarium pseudograminearum, F. graminearum, F. asiaticum, F. acuminatum, F. sinensis, F. equiseti, F. oxysporum, F. proliferatum, F. culmorum, and F. avenaceum. Fspp+Bs indicates Fusarium spp. and B. sorokiniana present in the same field; Fspp+Rc indicates Fusarium spp. and R. cerealis present in the same field; Fspp+Ggt indicates Fusarium spp. and G. graminis var. tritici present in the same field; Bs+Rc indicates B. sorokiniana and R. cerealis present in the same field; Bs+Ggt indicates B. sorokiniana and G. graminis var. tritici present in the same field; Rc+Ggt indicates R. cerealis and G. graminis var. tritici present in the same field; Fspp + Bs + Rc indicates Fusarium spp., B. sorokiniana, and R. cerealis present in the same field; Fspp+Bs+Ggt indicates Fusarium spp., B. sorokiniana, and G. graminis var. tritici present in the same field*.

Fungal combinations frequently coexisting in the same field and individual plants were *Fusarium* spp. - *B. sorokiniana, Fusarium* spp. - *R. cerealis, Fusarium* spp. - *G. graminis* var. *tritici, B. sorokiniana*- *R. cerealis, B. sorokiniana*- *G. graminis* var. *tritici*, and *R. cerealis*- *G. graminis* var. *tritici*. Among the combinations, *Fusarium* spp. - *B. sorokiniana* was the most frequent association (Tables 4, 5, Figure 2).

**Table 5:** Prevalence of *Fusarium* spp., *Bipolaris sorokiniana, Rhizoctonia cerealis*, and *Gaeumannomyces graminis* var. *tritici* or their combinations recovered from wheat roots or stems of plants sampled from 104 wheat fields from 2013 to 2016 in China.

**Species occurring in one sample (root or stem)**	**No. of roots**	**No. of stems**	**No. of plants**	**Percentage of plants (%)**
*Fspp*	381	713	690	36.3
*Bs*	257	433	446	23.4
*Rc*	8	71	55	2.9
*Ggt*	117	43	83	4.4
*Fspp + Bs*	73	116	220	11.6
*Fspp + Rc*	4	3	8	0.4
*Fspp + Ggt*	15	5	22	1.2
*Bs + Rc*	0	10	14	0.7
*Bs + Ggt*	15	16	54	2.8
*Rc + Ggt*	0	1	2	0.1
*Fspp + Bs + Rc*	0	1	4	0.2
*Fspp + Bs + Ggt*	2	2	15	0.8
Total	1,612	1,831	1,902	

*See Table 4. for abbreviations*.

### Correlations among climate variables and different species

There were clear relationships between the isolation frequency of a specific pathogen and MAP in the 12 cities, in this survey (Table 6). A negative correlation between the isolation frequency of *F. pseudograminearum* and MAP in the North China Plain was observed (Table 6). On the contrary, there was a positive correlation between the isolation frequency of *F. asiaticum* and the MAP during the period from 2013 to 2016. Moreover, a negative correlation between the isolation frequencies of *F. pseudograminearum* and MTCM was observed in the North China Plain (Table 6). There were clear relationships between the isolation frequencies of two pathogens in the 12 cities, in this survey (Table 6). A negative correlation between the isolation frequency of *F. pseudograminearum* and *G. graminis* var. *tritici* was observed in the North China Plain, while a positive correlation between the isolation frequencies of *F. sinensis* and *F. acuminatum* was observed (Table 6).

**Table 6:** Pearson pairwise correlations among climate and isolation frequency of a specific pathogen isolated from roots and stems in an area based on 4-year averages from 2013 to 2016.

	**MAT**	**MAP**	**MTCM**	**MTWM**	***pg***	***g***	***as*^b^**	***ac***	***s***	***Bs***	***Rc***
MAP	0.72^**^^a^	…	…	…	…	…	…	…	…	…	…
MTCM	0.91^**^	0.84^**^	…	…	…	…	…	…	…	…	…
MTWM	0.90^**^	0.45	0.68^*^	…	…	…	…	…	…	…	…
*pg*	−0.47	−0.71^**^	−0.64^*^	−0.35	…	…	…	…	…	…	…
*g*	−0.13	−0.05	−0.16	−0.05	−0.1	…	…	…	…	…	…
*as*	0.3	0.74^**^	0.44	0.04	−0.28	−0.26	…	…	…	…	…
*ac*	−0.01	0.23	0.14	−0.06	−0.49	−0.2	0.24	…	…	…	…
*s*	−0.46	−0.28	−0.32	−0.33	−0.22	−0.02	−0.18	0.75^**^	…	…	…
*Bs*	−0.49	−0.39	−0.49	−0.42	0.37	0.28	−0.29	−0.36	−0.1	…	…
*Rc*	0.3	−0.06	0.2	0.21	0.17	−0.27	0	−0.04	−0.35	−0.23	…
*Ggt*	0.1	0.48	0.2	0.08	−0.57^*^	0.41	0.28	0.36	0.11	0.12	−0.35

a “*” *indicates P < 0.05*,

“**” *indicates P < 0.01*.

b *Fusarium asiaticum (as), the other species see Table 2. for abbreviation*.

### Virulence of different species on wheat seedlings

All isolates of the seven *Fusarium* spp. and *B. sorokiniana* were able to cause seedling damping-off and/or rotting of the stem base (Figure 3 and Figure S1). *Fusarium pseudograminearum* proved to be the most severe pathogen and had a mean crown rot severity of 2.5–3.7 (mean = 3.1) (Figure 3). There was no significant difference in the mean crown rot severity between *F. pseudograminearum* and *B. sorokiniana* (mean = 2.3) or *F. equiseti* (mean = 2.1) (*P* < 0.05), although the mean crown rot severity of *F. pseudograminearum* isolates was higher than those of *F. graminearum, F. oxysporum, F. sinensis, F. acuminatum*, and *F. proliferatum* (*P* < 0.05) (Figure 3). *Fusarium oxysporum, F. acuminatum, F. sinensis*, and *F. proliferatum* exhibited weak aggressiveness on wheat seedlings and had a mean crown rot severity of 1.5, 1.2, 0.7, and 0.6, respectively.

## Discussion

This is the first report of a regional survey of wheat root and crown rot in the North China Plain. The findings presented here are partly in agreement with those of other regional studies conducted across the wheat growing regions of China (Li et al., 2011, 2012; Xu et al., 2014; Zhang et al., 2015). Li et al. (2012) first reported *F. pseudograminearum* causing FCR from Henan Province in China and *F. asiaticum* and *F. graminearum* causing wheat crown rot were found to be the predominant species in Henan, Hebei, and Shangdong in the North China Plain in a study conducted during 2009–2013 (Zhang et al., 2015). However, *F. pseudograminearum* was shown to be the predominant FCR pathogen in the North China Plain in this study. Although, *B. sorokiniana* associated with CRR was reported as an important pathogen of root and crown rot in wheat only in Jiangsu province of China (Li et al., 2011), it is now considered to be the major pathogen for this disease, with high field incidence (82.7%) and isolation frequency from roots (24%) and stems (33.7%) of wheat in the North China Plain (Tables 2, 3).

In this study, we found few isolates of *F. graminearum, F. asiaticum, F. acuminatum, F. sinensis, F. equiseti, F. oxysporum, F. proliferatum, F. culmorum, F. avenaceum*, and *R. cerealis*. In previous studies, these *Fusarium* spp. have been reported to cause wheat crown and foot rot in the PNW of the USA (Smiley and Patterson, 1996; Paulitz et al., 2002) and South Australia (Williams et al., 2002). Among the minor FCR pathogens, *F. graminearum, F. asiaticum, F. acuminatum, F. culmorum*, and *F. avenaceum* were already reported from China (Zhang et al., 2015; Ji et al., 2016; Li et al., 2016), while *F. sinensis, F. equiseti, F. oxysporum*, and *F. proliferatum* were first detected in this study. Especially, the *F. sinensis* isolates were found to be widely distributed and predominant in region 2. This is the first report of the association between *F. sinensis* and wheat FCR (Figure 1 and Figure S1). The ability of *F. sinensis* to cause FCR remains unknown when this species was first found in wheat seed and root samples, as reported by Zhao and Lu (2008). In contrast, in the present study, 113 *F. sinensis* isolates from wheat FCR were found in the North China Plain (Table 2, Figure 1). In addition, the *F. culmorum* isolates were the dominant pathogens in the cooler and higher rainfall areas of the PNW of the United States, the high rainfall region of Victoria in Australia, the South-East region of South Australia, and Turkey (Backhouse et al., 2004; Chakraborty et al., 2006; Tunali et al., 2008; Poole et al., 2013). Conversely, we recovered only one isolate of *F. culmorum* associated with wheat FCR in China (Table 2). Our data are in according to data of Li et al. (2016) that also recovered a single isolate of *F. culmorum*. Thus, this species does not appear to be as prevalent in China as in the other wheat-growing areas of the world.

We observed that the distribution frequency of *F. pseudograminearum* was higher in the low-rainfall areas in the North China Plain. The isolation frequency of *F. pseudograminearum* was higher in region 1 (462.2–546.5 mm annual rainfall during 2013–2016) and region 3 (564.2–587.6 mm annual rainfall during 2013–2016) compared to that in region 2 (520.7–752.4 mm annual rainfall during 2013–2016) and region 4 (732.2–1084.8 mm annual rainfall during 2013–2016) (Table 1, Figure 1). We detected a negative correlation between the frequency of occurrence of *F. pseudograminearum* and mean annual precipitation during 2013–2016 (*r* = −0.71; *P* < 0.01; Table 4). Similarly, *F. pseudograminearum* was predominant in the low-rainfall areas (with 250–500 mm rainfall) of the PNW of the USA (Smiley and Patterson, 1996; Poole et al., 2013), and Queensland and New South Wales in Eastern Australia (Akinsanmi et al., 2004). Other studies have suggested that the distribution of *F. pseudograminearum* was not only related to low rainfall, but also to raised temperatures in summer or elevated levels of carbon dioxide (Melloy et al., 2010; Moya-Elizondo et al., 2011).

In a previous study it was found that *F. asiaticum* and *F. graminearum*, causing wheat crown rot, were predominant in Henan, Hebei, and Shangdong in the North China Plain during 2009–2013 (Zhang et al., 2015). However, according to our results, the incidence of *F. graminearum* and *F. asiaticum* in the crown rot of wheat varied with the agronomic zone. *Fusarium graminearum* was predominant in regions 2 and 4 and was also scattered across region 1 (Figure 1). *Fusarium asiaticum* was found in region 4 (Figure 1). A positive correlation between the mean annual precipitation during 2013–2016 and the frequency of occurrence of *F. asiaticum* (*r* = 0.74; *P* < 0.01) was observed (Table 4).

Although *R. cerealis* is reported to be widespread in winter wheat in China (Chen et al., 2009), in our study, it was found to be more common in the wet areas, with the incidence being higher in regions 2 and 4 compared to that in regions 1 and 3 (Figure 1). Moreover, there was a significant difference in the incidence between regions 1 and 4.

FCR was once considered a component of common root rot, caused by *B. sorokiniana*, because of the occurrence of root rots caused by both pathogens in the same plant (Smiley and Patterson, 1996; Cook, 2010). Our results confirm this finding. The coexistence of *Fusarium* spp. and *B. sorokiniana* in the same field or in individual plants was more common than that of *Fusarium* spp. and *R. cerealis* or *G. graminis* var. *tritici* (Tables 4, 5, Figure 2). In previous studies, *R. cerealis* and *B. sorokiniana* or *F. culmorum* were found together from the group of stems in a particular field (Tunali et al., 2008). These results have also been confirmed in this study. However, we found other combinations of different species in the same field and in an individual plant. For example, *G. graminis* var. *tritici* and *B. sorokiniana* or *Fusarium* spp. were found to co-exist in the same field or in individual plants in this study (Tables 4, 5, Figure 2).

The results of seedling pathogenicity tests are in agreement with previous reports on the fungal pathogenicity of crowns or leaf sheath of wheat seedlings. *Fusarium pseudograminearum, F. graminearum*, and *B. sorokiniana* caused the damping-off of seedlings and rotted stem bases that resulted in the death of seedlings and were the most aggressive pathogens on wheat (Smiley and Patterson, 1996; Fernandez and Chen, 2005; Zhang et al., 2015). Other *Fusarium* strains that caused less severe FCR on wheat seedlings in greenhouse tests included some isolates of *F. equiseti, F. oxysporum, F. acuminatum*, and *F. proliferatum* (Akinsanmi et al., 2004; Fernandez and Chen, 2005; Chakraborty et al., 2006; Cook, 2010). However, in contrast to our results, *F. proliferatum* was observed to exhibit the same degree of aggressiveness as *F. pseudograminearum* and was more aggressive than *F. acuminatum* (Akinsanmi et al., 2004).

## Conclusion

Our results are in agreement with those of previously published studies wherein the pathogens of FCR, CRR, sharp eyespot, and take-all afflicting wheat were identified from China. However, our findings also demonstrate that the recovered species varied with the agronomic zone. The incidence of *F. pseudograminearum* and *R. cerealis* was significantly different between regions 1 and 4. A negative correlation was observed between the frequency of *F. pseudograminearum* occurrence and MAP during 2013–2016 in the North China Plain. We also observed *Fusarium* spp. - *B. sorokiniana* was the most frequent association. Moreover, for the first time, we report the association between *F. sinensis* and wheat FCR. Future research should focus on the current levels of resistance or tolerance to different species, which varied in the agronomic zones in local cultivars of the North China Plain. This work on species distribution has far-reaching implications that would maximize the efficiency of wheat breeding and management.

## Author contributions

YS and FX were responsible for coordinating the work (planning, implementation, and interpretation) and writing this manuscript. GY, JW, YL, and ZH conducted the fieldwork and were involved in the survey and sampling. KZ conducted the laboratory work and was involved in the isolation, culture, and identification of species. LL was responsible for all DNA analyses.

## Conflict of interest statement

The authors declare that the research was conducted in the absence of any commercial or financial relationships that could be construed as a potential conflict of interest.

## Supplementary material

Figure S1 Colony morphology on potato dextrose agar (PDA) (25°C, darkness, 6 days), pathogenicity of isolates G14LY24-2 (*Fusarium pseudograminearum*), G13YY1-4-2 (*F. graminearum*), G13XX2-3 (*F. acuminatum*), G13AY1-25 (*F. sinensis*), G13LH3-46-3 (*F. equiseti*), G14WX2-19-2 (*F. oxysporum*), G13AY1-4-2 (*F. proliferatum*), and G14LY24-4-1 (*Bipolaris sorokiniana*) on wheat seedlings (*Triticum aestivum* cultivar “Zhengmai 366”) in a glasshouse with a day/night photoperiod of 12/12 h at a temperature of 25/15°C and relative humidity of 60/80 (±5) % at day 35 after inoculation.

Table S1 Prevalence of *Fusarium pseudograminearum, F. graminearum, F. asiaticum, F. acuminatum*, and *F. sinensis* or combinations recovered from wheat plants sampled from 104 wheat fields in 2013–2016 in China.

Table S2 Plant numbers of mixed infection types of different *Fusarium* spp.

Table S3 GenBank Accession numbers (partial translation elongation factor-1α gene sequences) of *Fusarium* spp. isolated form wheat roots and stems from the North China Plain in 2013–2016.
