# Supplementary figures and images for: Spatial Distribution of Root and Crown Rot Fungi Associated With Winter Wheat in the North China Plain and Its Relationship With Climate Variables
Source: Front Microbiol. 2018 May 25;9:1054. doi: 10.3389/fmicb.2018.01054 (PMC5981207; doi:10.3389/fmicb.2018.01054)

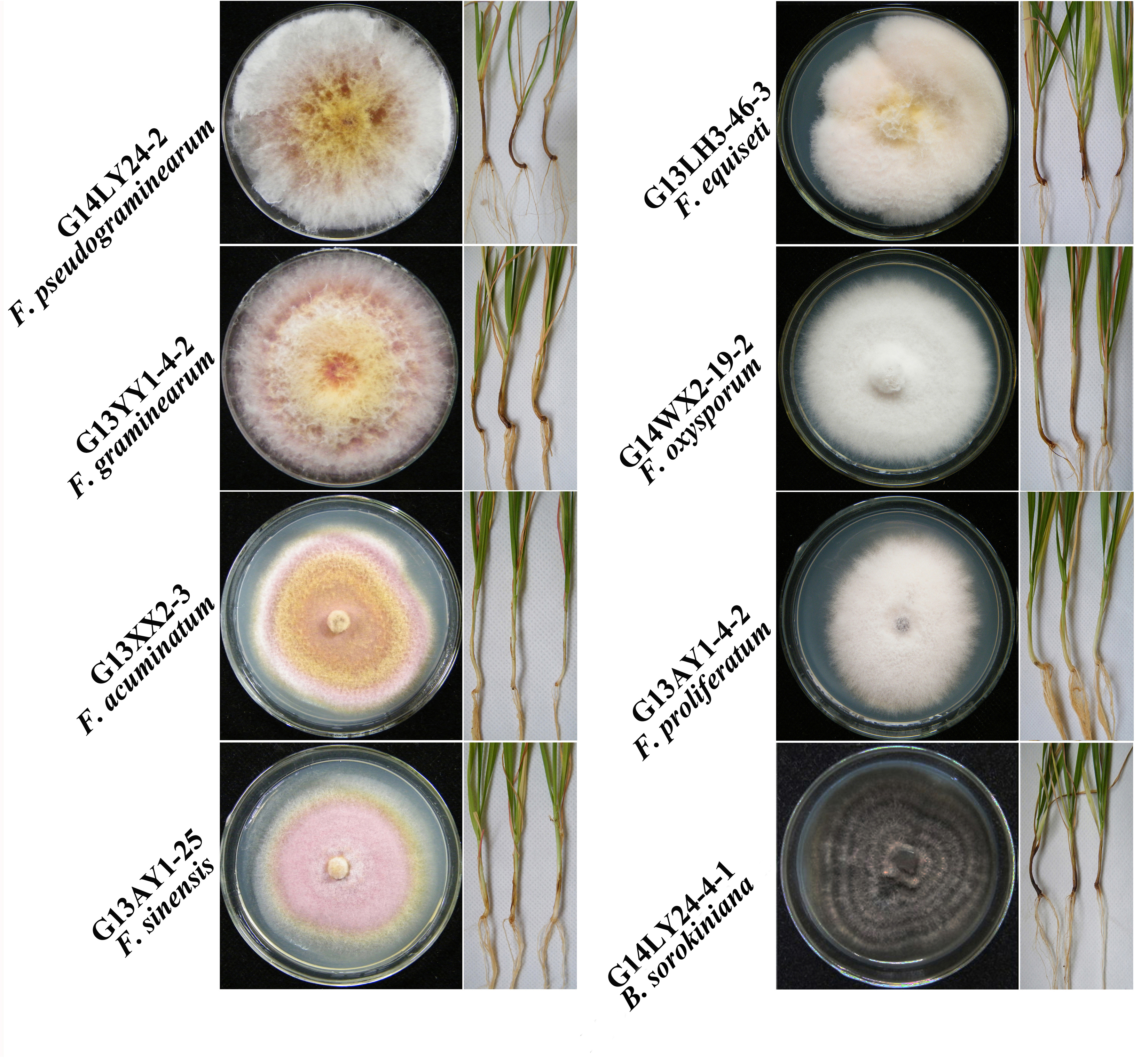

Supplement: Figure S1 — Colony morphology on potato dextrose agar (PDA) (25°C, darkness, 6 days), pathogenicity of isolates G14LY24-2 (Fusarium pseudograminearum), G13YY1-4-2 (F. graminearum), G13XX2-3 (F. acuminatum), G13AY1-25 (F. sinensis), G13LH3-46-3 (F. equiseti), G14WX2-19-2 (F. oxysporum), G13AY1-4-2 (F. proliferatum), and G14LY24-4-1 (Bipolaris sorokiniana) on wheat seedlings (Triticum aestivum cultivar “Zhengmai 366”) in a glasshouse with a day/night photoperiod of 12/12 h at a temperature of 25/15°C and relative humidity of 60/80 (±5) % at day 35 after inoculation. [file Image_1.JPEG]
