# Supplementary material for: Spatial Distribution of Root and Crown Rot Fungi Associated With Winter Wheat in the North China Plain and Its Relationship With Climate Variables
Source: Front Microbiol. 2018 May 25;9:1054. doi: 10.3389/fmicb.2018.01054 (PMC5981207; doi:10.3389/fmicb.2018.01054)
Supplement: Table S1 — Prevalence of Fusarium pseudograminearum, F. graminearum, F. asiaticum, F. acuminatum, and F. sinensis or combinations recovered from wheat plants sampled from 104 wheat fields in 2013–2016 in China. [file Table_1.DOC]

Table S1. Prevalence of *Fusarium pseudograminearum*, *F. graminearum*, *F. asiaticum*, *F. acuminatum* and *F. sinensis* or combinations recovered from wheat plants sampled from 104 wheat fields in 2013-2016 in China.

| Species types ^a^ | Percentage of fields with species present (%) |
| --- | --- |
| *pg* | 49.0 |
| *g* | 20.2 |
| *as* | 3.8 |
| *ac* | 26.0 |
| *s* | 29.8 |
| *pg+g* | 11.5 |
| *pg+as* | 2.9 |
| *pg+ac* | 9.6 |
| *pg+s* | 10.6 |
| *g+as* | 1.0 |
| *g+ac* | 5.8 |
| *g+s* | 7.7 |
| *as+ac* | 1.9 |
| *ac+s* | 13.5 |
| *pg*+*g*+*as* | 1.0 |
| *pg*+*g*+*ac* | 1.9 |
| *pg*+*g*+*s* | 2.9 |
| *pg*+*as*+*ac* | 1.0 |
| *pg*+*ac*+*s* | 2.9 |
| *g*+*ac*+*s* | 2.9 |

^a^*Fusarium pseudograminearum* (*pg*), *F. graminearum* (*g*), *F. asiaticum* (*as*), *F. acuminatum* (*ac*), *F. sinensis* (*s*).
