# Supplementary material for: Spatial Distribution of Root and Crown Rot Fungi Associated With Winter Wheat in the North China Plain and Its Relationship With Climate Variables
Source: Front Microbiol. 2018 May 25;9:1054. doi: 10.3389/fmicb.2018.01054 (PMC5981207; doi:10.3389/fmicb.2018.01054)
Supplement: Table S2 — Plant numbers of mixed infection types of different Fusarium spp. [file Table_2.DOC]

Table S2. Plant numbers of mixed infection types of different *Fusarium* spp.^a^ from wheat plants.

| Field | Location | Year | Mixed infection type (Plant number) |
| --- | --- | --- | --- |
| G16SX3-2 | Luoyang | 2016 | *pg*+*e*(1) |
| G16WX7 | Jiaozuo | 2016 | *pg*+*e*(2), *pg*+*pr*(1) |
| G16NH2-2 | Anyang | 2016 | *pg*+*o*(1), *g*+*pr*(1) |
| G16FX3 | Puyang | 2016 | *pg*+*ac*(1), *pg*+*as*(1), *pg*+*pr*(1) |
| G15NH2 | Anyang | 2015 | *pg+c*(1), *pg+g*(1) |
| G15SMX1 | Sanmenxia | 2015 | *ac+av*(1), *ac+s*(2) |
| G15XUNX1 | Hebi | 2015 | *pg+ac*(2), *pg+s*(2) |
| G14WX2 | Jiaozuo | 2014 | *ac+e*(1), *e+o*(1) |
| G14XY4 | Xinyang | 2014 | *g+ac*(2) |
| G13AY1 | Anyang | 2013 | *g+ac*(1) |
| G13AY2 | Anyang | 2013 | *ac+s*(1) |
| G13HB1 | Hebi | 2013 | *pg+ac*(1), *pg+s*(1), *g+s*(1) |
| G13LH2 | Luohe | 2013 | *g+s*(1) |
| G13LH3 | Luohe | 2013 | *s+e*(1) |
| G13WX2 | Jiaozuo | 2013 | *pg+s*(1) |
| G13XX2 | Xinxiang | 2013 | *g+ac*(1) |
| G13YJ1 | Xinxiang | 2013 | *ac+e*(1), *ac+o*(1) |

^a^*Fusarium pseudograminearum* (*pg*), *F. graminearum* (*g*), *F. acuminatum* (*ac*), *F. sinensis* (*s*), *F. equiseti* (*e*), *F. oxysporum* (*o*), *F. culmorum* (*c*), *F. avenaceum* (*av*), *F. proliferatum* (*pr*).
