# Supplementary material for: Spatial Distribution of Root and Crown Rot Fungi Associated With Winter Wheat in the North China Plain and Its Relationship With Climate Variables
Source: Front Microbiol. 2018 May 25;9:1054. doi: 10.3389/fmicb.2018.01054 (PMC5981207; doi:10.3389/fmicb.2018.01054)
Supplement: Table S3 — GenBank Accession numbers (partial translation elongation factor-1α gene sequences) of Fusarium spp. isolated form wheat roots and stems from the North China Plain in 2013–2016. [file Table_3.doc]

Table S3. GenBank Accession numbers (partial translation elongation factor-1α gene sequences) of *Fusarium* spp. isolated form wheat roots and stems from the North China Plain in 2013-16.

| Field | Year | Location | GenBank Accession number |
| --- | --- | --- | --- |
| G13AY1 | 2013 | Anyang, Henan province | KX663591-KX663598 |
| G13AY2 | 2013 | Anyang, Henan province | KX663599- KX663603 |
| G13HB1 | 2013 | Hebi, Henan province | KX663604- KX663620 |
| G13HB2 | 2013 | Hebi, Henan province | KX663621- KX663622 |
| G13HX1 | 2013 | Anyang, Henan province | KX663623- KX663630 |
| G13LH1 | 2013 | Luohe, Henan province | KX663631-KX663644 |
| G13LH2 | 2013 | Luohe, Henan province | KX663645-KX663657 |
| G13LH3 | 2013 | Luohe, Henan province | KX663658-KX663677 |
| G13NX1 | 2013 | Nanyang, Henan province | KX663678 |
| G13NX2 | 2013 | Nanyang, Henan province | KX663679 |
| G13NX3 | 2013 | Nanyang, Henan province | KX663680- KX663684 |
| G13PY1 | 2013 | Puyang, Henan province | KX663685- KX663691 |
| G13PY2 | 2013 | Puyang, Henan province | KX663692-KX663708 |
| G13WX1 | 2013 | Jiaozuo, Henan province | KX663709-KX663735 |
| G13WX2 | 2013 | Jiaozuo, Henan province | KX663736- KX663765 |
| G13XX1 | 2013 | Xinxiang, Henan province | KX663767- KX663787 |
| G13XX2 | 2013 | Xinxiang, Henan province | KX663788- KX663801 |
| G13XY1 | 2013 | Nanyang, Henan province | KX663802-KX663805 |
| G13YJ1 | 2013 | Xinxiang, Henan province | KX663806- KX663822 |
| G13YY1 | 2013 | Xinxiang, Henan province | KX663823 |
| G14AY4 | 2014 | Anyang, Henan province | KX702405- KX702406 |
| G14KF3 | 2014 | Kaifeng, Henan province | KX702407- KX702408 |
| G14LY9 | 2014 | Luoyang, Henan province | KX702409 |
| G14LY14 | 2014 | Luoyang, Henan province | KX702410- KX702412 |
| G14LY20 | 2014 | Luoyang, Henan province | KX702413 |
| G14LY24 | 2014 | Luoyang, Henan province | KX702414-KX702469 |
| G14LZ1 | 2014 | Anyang, Henan province | KX702470-KX702487 |
| G14LZ2 | 2014 | Anyang, Henan province | KX702488- KX702490 |
| G14PY1 | 2014 | Puyang, Henan province | KX702491- KX702494 |
| G14WX1 | 2014 | Jiaozuo, Henan province | KX702495- KX702525 |
| G14WX2 | 2014 | Jiaozuo, Henan province | KX702526- KX702537 |
| G14XX1 | 2014 | Xinxiang, Henan province | KX702538-KX702555 |
| G14XY4 | 2014 | Xinyang, Henan province | KX702556- KX702563 |
| G14YJ1 | 2014 | Xinxiang, Henan province | KX702565- KX702573 |
| G14YJ2 | 2014 | Xinxiang, Henan province | KX702574-KX702576 |
| G14YJ3 | 2014 | Xinxiang, Henan province | KX702577- KX702579 |
| G14YY2 | 2014 | Xinxiang, Henan province | KX702580- KX702581 |
| G14YY3 | 2014 | Xinxiang, Henan province | KX702582- KX702586 |
| G14YY4D | 2014 | Xinxiang, Henan province | KX702587- KX702595 |
| G14YY6D | 2014 | Xinxiang, Henan province | KX702596 |
| G14ZK3 | 2014 | Zhoukou, Henan province | KX702597 |
| G15FQ1 | 2015 | Xinxiang, Henan province | KX702598 |
| G15FQ2 | 2015 | Xinxiang, Henan province | KX702599- KX702605 |
| G15HB1 | 2015 | Hebi, Henan province | KX702606-KX702612 |
| G15HX1 | 2015 | Anyang, Henan province | KX702613 |
| G15HX2 | 2015 | Anyang, Henan province | KX702614 |
| G15KF1 | 2015 | Kaifeng, Henan province | KX702615-KX702616 |
| G15KF3 | 2015 | Kaifeng, Henan province | KX702617- KX702618 |
| G15LZ1 | 2015 | Anyang, Henan province | KX702619- KX702623 |
| G15LZ2 | 2015 | Anyang, Henan province | KX702624 |
| G15NH2 | 2015 | Anyang, Henan province | KX702625-KX702689 |
| G15NH3 | 2015 | Anyang, Henan province | KX702690- KX702697 |
| G15PY3 | 2015 | Puyang, Henan province | KX702699-KX702708 |
| G15SMX1 | 2015 | Sanmenxia, Henan province | KX702709- KX702720 |
| G15SX1 | 2015 | Luoyang, Henan province | KX702721-KX702731 |
| G15WX2 | 2015 | Jiaozuo, Henan province | KX702732- KX702742 |
| G15WZ1 | 2015 | Jiaozuo, Henan province | KX702743- KX702748 |
| G15XUNX1 | 2015 | Hebi, Henan province | KX702749-KX702786 |
| G15XX2 | 2015 | Xinxiang, Henan province | KX702787- KX702799 |
| G15YY1 | 2015 | Xinxiang, Henan province | KX702800 |
| G16FQ4 | 2016 | Xinxiang, Henan province | KY081469- KY081480 |
| G16FX3 | 2016 | Puyang, Henan province | KY081481- KY081490 |
| G16FX4 | 2016 | Puyang, Henan province | KY081491- KY081501 |
| G16FX5 | 2016 | Puyang, Henan province | KY081502- KY081513 |
| G16HBS1 | 2016 | Handan,Hebei province | KY081514- KY081517 |
| G16HX1 | 2016 | Anyang, Henan province | KY081518- KY081524 |
| G16LH1 | 2016 | Luohe, Henan province | KY081525-KY081529 |
| G16NH2-2 | 2016 | Anyang, Henan province | KY081530- KY081558, KY081566-KY081572 |
| G16NH2-3 | 2016 | Anyang, Henan province | KY081559- KY081565 |
| G16NY1 | 2016 | Nanyang, Henan province | KY081573 |
| G16NY2 | 2016 | Nanyang, Henan province | KY081574, KY081586 |
| G16NY2-2 | 2016 | Nanyang, Henan province | KY081575- KY081585 |
| G16SDS1 | 2016 | Heze, Shandong province | KY081587- KY081589 |
| G16SX2 | 2016 | Luoyang, Henan province | KY081590 |
| G16SX3-2 | 2016 | Luoyang, Henan province | KY081591-KY081594 |
| G16TH6 | 2016 | Nanyang, Henan province | KY081595- KY081596 |
| G16WX7 | 2016 | Jiaozuo, Henan province | KY081597- KY081609 |
| G16WX8 | 2016 | Jiaozuo, Henan province | KY081610 |
| G16XY1 | 2016 | Xinyang, Henan province | KY081613- KY081614 |
| G16YJ1 | 2016 | Xinxiang, Henan province | KY081615-KY081619 |
| G16YY5 | 2016 | Xinxiang, Henan province | KY081620- KY081636 |
